# Food allergy and anaphylaxis – 2037. Emergency department management of insect-sting allergic reactions in a community hospital in the United States

**DOI:** 10.1186/1939-4551-6-S1-P122

**Published:** 2013-04-23

**Authors:** M Razi Rafeeq, Manoj Dhariwal

**Affiliations:** 1 Allergy and Immunology, Private Practice, Oregon, OH, USA; 2 Flower Hospital, Sylvania, OH, USA

## Background

A general consensus on the definition and clinical diagnostic criteria for anaphylaxis has been reached and recommended national and global guidelines for its management have been published. However, few studies in the United States have examined the extent to which emergency management of insect-sting allergic reactions adheres to these guidelines. The objective of this project was to evaluate the management of insect-sting allergic reactions, including those resulting in anaphylaxis, in a community hospital emergency department in order to measure adherence to the published national guidelines.

## Methods

This project involved the retrospective review of all records of patients who were treated for acute allergic reactions to insect venom (ICD-9-CM diagnosis code 989.5) in the emergency department of a community hospital between April 2009 and April 2011.

## Results

A total of 133 records were identified between April 2009 and April 2011. Of these, 81 patients experienced local reactions while 52 were systemic reactions. Of the 52 patients experiencing systemic reactions, 45 were classified as anaphylaxis as defined by the NIAID-FAAN criteria. Of the 45 patients classified as anaphylaxis, 6 patients received epinephrine administered by the emrgency medical services (EMS) before arrival and an additional 10 received epinephrine while in the emergency department, such that 35% of patients received epinephrine. Other recommended interventions included H1 antihistamines (64%), H2 antihistamines (60%), and corticosteroids (80%). A prescription for self-injectable epinephrine at the time of discharge was documented for 33% of patients with anaphylaxis. None of the patients had documentation of referral to an allergy specialist.

## Conclusions

In a community hospital emergency department in the United States, adherence to the national guideline-recommended management of patients presenting with insect-sting-related anaphylaxis is low. Intensive efforts and creative programs to educate physicians and other healthcare professionals in identification and proper management of anaphylaxis are necessary to improve treatment. Development of an electronic record template for quick identification of symptoms and corresponding recommended management may help improve adherence to national guidelines.

